# Sorting the Wheat from the Chaff: Identifying miRNAs in Genomic Survey Sequences of *Triticum aestivum* Chromosome 1AL

**DOI:** 10.1371/journal.pone.0040859

**Published:** 2012-07-17

**Authors:** Stuart J. Lucas, Hikmet Budak

**Affiliations:** Faculty of Engineering and Natural Sciences, Sabanci University, Orhanlı, Istanbul, Turkey; Nottingham University, United Kingdom

## Abstract

Individual chromosome-based studies of bread wheat are beginning to provide valuable structural and functional information about one of the world’s most important crops. As new genome sequences become available, identifying miRNA coding sequences is arguably as important a task as annotating protein coding sequences, but one that is not as well developed. We compared conservation-based identification of conserved miRNAs in 1.5× coverage survey sequences of wheat chromosome 1AL with a predictive method based on pre-miRNA hairpin structure alone. In total, 42 sequences expected to encode conserved miRNAs were identified on chromosome 1AL, including members of several miRNA families that have not previously been reported to be expressed in T. aestivum. In addition, we demonstrate that a number of sequences previously annotated as novel wheat miRNAs are closely related to transposable elements, particularly Miniature Inverted Terminal repeat Elements (MITEs). Some of these TE-miRNAs may well have a functional role, but separating true miRNA coding sequences from TEs in genomic sequences is far from straightforward. We propose a strategy for annotation to minimize the risk of mis-identifying TE sequences as miRNAs.

## Introduction

Bread wheat (*Triticum aestivum* L.) is arguably the world’s most important crop plant, occupying 17% of all cultivated land and supplying about 55% of all carbohydrates [1], but its large (∼16 GB) genome has not yet been sequenced owing to its complex and repetitive nature. *T. aestivum* is a hexaploid believed to derive from serial hybridization events between three different diploid wheat ancestors [2]. For this reason each of its 7 chromosomes is present in 3 phylogenetically related but divergent sub-genomes (formula AABBDD, 6 n = 42). However, using newly developed chromosome sorting techniques [3], individual chromosomes can be studied, resolving the problem of identifying which sub-genome a particular feature belongs to. Projects are now underway to carry out initial survey sequencing of each bread wheat chromosome using new-generation sequencing platforms (International Wheat Genome Sequencing Consortium, www.wheatgenome.org), and these studies are already revealing valuable information about wheat genome structure [4], [5].

MicroRNAs (miRNAs) are small, non-coding single-stranded RNA molecules whose primary function is regulation of gene expression at the post-transcriptional level [6]. Plant miRNA genes are generally independent of protein-coding genes, and produce a long primary transcript (pri-miRNA) that then undergoes 2 cleavage events, the first giving a precursor (pre-miRNA) that folds into a hairpin structure, the second extracting the mature 19–24 nt miRNA from the stem of the hairpin (recently reviewed in [7]). The mature miRNA sequence may be found in either side of the hairpin and is excised along with its complementary sequence in a duplex with 2-nucleotide 3′ overhangs [8]. The duplex is unwound and the miRNA preferentially incorporated into the RNA-induced silencing complex (RISC), where it directs transcriptional repression of cognate mRNA targets [9]. The complementary sequence (referred to as miRNA*) was generally thought to be degraded, but recent evidence suggests that in many cases it may also be functional [10].

In just 10 years since the first plant miRNA was identified [11], this mechanism has been recognized to be ubiquitous in plants and important in an increasing variety of biological processes, including development, their own biogenesis, and biotic and abiotic stress responses (reviewed in [12], [13]). In view of this miRNA identification studies have been carried out in a growing number of plant species, either by sequencing of small RNA libraries or bioinformatic analysis [14]. However, miRNA identification in plants is complicated by the fact that, unlike animals, they are not the dominant small RNA species, but one element in a mixture of different types of small RNAs [9]. Most abundant are ‘small inhibitory’ siRNAs, which resemble miRNAs in size and their ability to recruit the RISC to degrade RNA, but are derived from double-stranded RNAs and thus restrict the replication of plant viruses and transposable elements [15]. Therefore, criteria have been established for the accurate annotation of plant miRNAs [8]. Deep sequencing of cDNA libraries generated from small RNAs is the most powerful experimental method available for identifying novel plant miRNAs; however, to ensure that the candidates are miRNAs rather than other small RNA types, their pre-miRNA hairpins must also be detected in ESTs or genomic sequences. This approach has been used to discover miRNAs in pooled tissues of *T. aestivum* [16], [17] as well as wheat leaves subjected to powdery mildew infection or heat stress [18]. A less resource-intensive but still effective means of finding miRNAs is bioinformatic analysis, exploiting the high conservation of miRNAs between plant species. This has the advantage of being able to identify miRNAs that are only expressed at very low levels, or under very specific conditions. In this strategy, similarity searches are first used to identify candidate paralogs to known mature miRNAs; the sequences these are found in are then analyzed to test whether they have the characteristics of pre-miRNA hairpins, discussed in detail in [14]. Finally, to be confirmed as a miRNA the candidate must have been verified experimentally in at least one other plant species [8]. This approach was used to identify the first miRNAs in ESTs from several wild relatives of bread wheat [19] as well as the closely related model grass species *Brachypodium distachyon* [20]. Expression of many of these conserved miRNAs has since been verified using a plant miRNA microarray, which also enabled changes in expression in response to stress to be assessed [21], [22]. The disadvantage of detecting miRNAs by conservation is that it is not possible to discover novel species-specific miRNAs. Therefore a number of groups are developing miRNA prediction tools, using support vector machine (SVM) algorithms to identify plant pre-miRNA sequences based on the empirically derived characteristics of a training set of known pre-miRNA sequences (e.g. [23], [24]).

Increasing amounts of genomic sequence data will become available for different crop species over the next few years. Given the broad functional significance of miRNAs, identification of miRNA coding sequences is arguably as important as predicting protein-coding genes, but much less well developed. In this study, we utilize survey sequences of the 523 MB long arm of *T. aestivum* chromosome 1A (1AL) to compare *in silico* miRNA identification, by sequence conservation-based and predictive methods. In doing so we generate a catalogue of putative miRNA genes present on chromosome, which is compared with the miRNAs known to be expressed in bread wheat.

## Results

### Analysis of Conserved Sequences Identifies miRNA Coding Sequences in 1AL

#### Script development and prediction criteria

When searching computationally for putative miRNA sequences in a large plant genome such as wheat, there is a significant risk of generating false positives from non-miRNA inverted repeat sequences, and getting the right balance between selectivity and sensitivity is difficult [25]. Chromosome 1AL consists of up to 90% repetitive elements [26], including many inverted repeat regions that could form miRNA-like hairpin structures. Therefore while developing automated scripts to find conserved miRNAs in chromosome 1AL, we decided to use relatively stringent limits and cut-offs from the range of recommendations found in the literature. During initial similarity searches, only hits with 2 or fewer mismatches to a known mature miRNA sequence were retained. When using default parameters, BLAST awards a higher score to shorter sequences that are identical to most of the query, than to those which cover the full length of the query but include a mismatch within them. As conserved miRNA sequences are as likely to contain internal mismatches as at their ends, our first script, SUmirFind (Methods S1) modifies the BLAST parameters to avoid this bias and adds the absent bases in shorter hits to the calculation of the number of mismatches.

From a total of 2,048,861 1AL sequence reads with an average length of 532 bases, 11,041 contained sequences with 2 or fewer mismatches to a published mature miRNA. These sequences were then checked to see whether they form pre-miRNA-like hairpin structures in a 2 step process using our second script, SUmirFold (Materials & Methods and Methods S1). Putative pre-miRNA sequences were selected using the following criteria: the miRNA:miRNA* duplex is considered to start 2 bp before the 5′ end of the mature miRNA sequence, to include the 3′-overhang of the miRNA*. Within this extended duplex, no more than 4 nucleotides in the putative miRNA may be unpaired [8]; the miRNA* strand of this duplex must be no more than 3 nt longer than the miRNA strand (eliminating structures where the miRNA* is broken into separate segments, or contains a large loop); the mature miRNA sequence must not go round the head of the hairpin; the hairpin must have a GC content between 24–71% and a Minimum Free Energy Index (MFEI) >0.67. These last two criteria help discriminate between miRNAs and other ssRNA species such as tRNA and rRNA [14]. In addition, cases where the putative miRNA:miRNA* is perfectly base-paired are more likely to be inverted repeats than miRNA genes [25], so these instances are marked as suspect and placed in a separate results table and folder.

#### Identification of conserved miRNAs in chromosome 1AL

The number of possible pre-miRNA hits passing these criteria was 3095, while a further 484 suspect sequences passed all but the final criterion. When two or more query miRNAs differing by less than 2 nucleotides both matched the same sequence, all but the best match were eliminated. In addition, as the 1AL genomic survey sequences give only 1.5× coverage [4], any miRNA represented by 10 or more putative hits was considered likely to be matching repeat sequences; these were removed to be analyzed separately (below). Finally, the predicted secondary structures of the remaining sequences in the ‘suspect’ table (only 11 by this point) were examined individually, and transferred to the results table if appropriate. Following these data analysis steps, 42 putative pre-miRNA coding sequences were predicted with high confidence from the 1AL survey sequences, representing 20 different miRNA and 1 miRNA* species (Tables 1 and 2; full details including pre-miRNA sequences are in Table S1). The 3 sequences containing miR166 family members all gave matches to both the miRNA and miRNA* in opposite arms of the same hairpin. Also, 4 sequences contained two adjacent hairpins that both passed the pre-miRNA criteria; in each of these cases the 2 hits were to members of the same miRNA family (1 pair each for miR156, miR1121, miR2118 and miR5050), and so may represent tandem repeats of these miRNAs. To look for evidence of expression, the putative pre-miRNA sequences were used to search the wheat EST database at www.ncbi.nlm.nih.gov. To avoid mis-identifying ESTs transcribed from homologous miRNA loci on other chromosomes, only matches of >98% identity across the entire pre-miRNA were accepted as evidence of expression from 1AL. By this criterion 8 pre-miRNAs were shown to be expressed – for miR171a, miR393a, miR399b (two different pre-miRNAs), miR5050 (2 different pre-miRNAs), miR5075 and miR5200, demonstrating that these miRNAs are expressed from chromosome 1AL (Table 1). Most of the other high-confidence miRNAs found here also matched ESTs, but at lower sequence identity.

**Table 1:** Conserved miRNAs shown to be expressed from *T. aestivum* chromosome 1AL.

miRNA from chromosome 1AL				Conserved miRNA^1^	Mature miRNA location			Pre-miRNA statistics				Matched sequence read^2^
ID	Sequence^3^	Length(nt)		ID	Start	End	Arm	Length	MFE^4^	GC%	MFEI^5^	ID
tae-miR171a	UGAUUGAGCCGCGCCAAUAU		20	zma-miR171a	78	97	3′	118	−59	48.31	1.04	**F003IAL01BBGXU**
tae-miR393a	UCCAAAGGGAUCGCAUUGAUCC		22	bdi-miR393a	20	41	5′	130	−66	53.08	0.95	F2MIQBM01BALL6
tae-miR399b	UGCCAAAGGAGAAUUGCCCUG		21	bdi-miR399b	120	140	3′	161	−64	57.14	0.69	F1NBZEY01AK17M
tae-miR399b	UGCCAAAGGAGAAUUGCCCUG		21	bdi-miR399b	111	131	3′	152	−64	57.89	0.73	F1NBZEY02GW67Y
tae-miR5075	**GC**CUCCGUCGCCGCCGUCCGC		21	osa-miR5075	20	40	5′	308	−147	69.16	0.69	F0RUNSI01CTDLK
tae-miR5050	**A**UGAGGUCGUUCAACCAGCAA		21	hvu-miR5050	92	112	3′	133	−72	60.90	0.89	**F1ADE5F01D2FWK**
tae-miR5050	**G**UGAGGUCGUUCAAC**C**GGCAA		21	hvu-miR5050	92	112	3′	133	−75	60.90	0.92	**F1ADE5F01D2FWK**
tae-miR5200	UGUAGAUACUC**C**CUAAGGCUU		21	bdi-miR5200	76	96	3′	117	−39	38.46	0.86	**F2MIQBM01ARRO6**

1 Where two similar known miRNAs gave equally close matches to a sequence, the evolutionarily closest match is given.

2 Matched sequence reads shown in bold were also predicted to form miRNA hairpins by miRPara.

3 Mismatches to the conserved miRNA sequence are underlined and in bold.

4 MFE  =  Minimum Folding free Energy of predicted hairpin secondary structure.

5 MFEI  =  Minimum Folding Energy Index, calculated as described by Yin et al. [40].

**Table 2:** High-confidence predicted miRNA coding sequences on chromosome 1AL.

miRNA from chromosome 1AL				Conserved miRNA^1^	Mature miRNA location			Pre-miRNA statistics				Matched sequence read^2^
ID	Sequence^3^	Length(nt)		ID	Start	End	Arm	Length	MFE^4^	GC%	MFEI^5^	ID
tae-miR156a	UGACAGAAGAGAGUGAGCAC		20	aly-miR156a	20	39	5′	125	−62	53.60	0.93	**F2MIQBM02DWQ3H**
tae-miR156a	UGACAGAAGAGAGUGAGCAC		20	aly-miR156a	20	39	5′	124	−69	58.87	0.95	**F2MIQBM02DWQ3H**
tae-miR164a	UGGAGAAGCAGGGCACGUGCA		21	aly-miR164a	20	40	5′	140	−75	55.71	0.96	**F0RUNSI02GP0XY**
tae-miR164a	UGGAGAAGCAGGGCACGUGCA		21	aly-miR164a	20	40	5′	140	−75	55.71	0.96	**F1NBZEY01BCHIA**
tae-miR166b*	GGAAUGUUGUCUGGUUCAAGG		21	zma-miR166b*	20	40	5′	136	−55	46.32	0.87	**F1ADE5F01D77GU**
tae-miR166e	CUCGGACCAGGCUUCAUUCCC		21	bdi-miR166e	94	114	3′	As above				**F1ADE5F01D77GU**
tae-miR166a	UCGGACCAGGCUUCAUUCCCC		21	aly-miR166a	95	115	3′	136	−54	47.06	0.84	**F1ADE5F01DOFSZ**
tae-miR166b*	GGAAUGUUGUCUGGUUCAAGG		21	zma-miR166b*	20	40	5′	As above				**F1ADE5F01DOFSZ**
tae-miR166a	UCGGACCAGGCUUCAUUCCCC		21	aly-miR166a	96	116	3′	137	−55	46.72	0.86	**F1ADE5F01E2LUR**
tae-miR166b*	GGAAUGUUGUCUGGUUCAAGG		21	zma-miR166b*	20	40	5′	As above				**F1ADE5F01E2LUR**
tae-miR171a	UGAUUGAGCCGCGCCAAUAU		20	zma-miR171a	78	97	3′	118	−59	48.31	1.04	**F003IAL01EKU7V**
tae-miR171b	UUGAGCCGUGCCAAUAUCAC		20	zma-miR171b	82	101	3′	122	−59	59.02	0.82	**F1ADE5F01DL80Q**
tae-miR172a	AGAAUCUUGAUGAUGCUGCA		20	csi-miR172a	133	152	3′	173	−74	45.09	0.94	F2MIQBM01BUBWA
tae-miR172a	AGAAUCUUGAUGAUGCUGCA		20	csi-miR172a	133	152	3′	173	−73	45.66	0.92	F1NBZEY02GWGQ1
tae-miR172a	AGAAUCUUGAUGAUGCUGCA		20	csi-miR172a	134	153	3′	174	−69	45.98	0.86	F1ADE5F01BAC6G
tae-miR172a	AGAAUCUUGAUGAUGCUGCA		20	csi-miR172a	136	155	3′	176	−70	44.32	0.90	F1ADE5F01AFV6T
tae-miR172a	AGAAUCUUGAUGAUGCUGCA		20	csi-miR172a	135	154	3′	175	−73	44.00	0.95	F1ADE5F01DARNZ
tae-miR172a	AGAAUCUUGAUGAUGCUGCA		20	csi-miR172a	136	155	3′	176	−74	44.32	0.95	F1ADE5F01AJ0FQ
tae-miR172a	AGAAUCUUGAUGAUGCUGCA		20	csi-miR172a	133	152	3′	173	−69	45.66	0.87	F1ADE5F01BL63C
tae-miR172a	AGAAUCUUGAUGAUGCUGCA		20	csi-miR172a	136	155	3′	176	−70	43.75	0.91	F1ADE5F01AY2DE
tae-miR399b	UGCCAAAGGAGAAUUGCCCUG		21	bdi-miR399b	100	120	3′	141	−64	59.57	0.76	**F1NBZEY01CXBWQ**
tae-miR399b	UGCCAAAGGAGAAUUGCCCUG		21	bdi-miR399b	139	159	3′	180	−84	59.44	0.78	F2MIQBM01AUXGN
tae-miR399b	UGCCAAAGGAGAAUUGCCCUG		21	bdi-miR399b	138	158	3′	179	−84	59.22	0.79	F2MIQBM01B24OQ
tae-miR399k	UGCCAAAGGAAAUUUGCCCC**A**		21	osa-miR399k	93	113	3′	134	−54	58.96	0.68	F1ADE5F01C5M0T
tae-miR1138	G**U**UUAGAUGUGACAUCCUUAAAA		23	tae-miR1138	20	42	5′	173	−57	32.37	1.01	F0RUNSI01BXR55
tae-miR2118g	UUCCUAAUGCCUCCCAUUCCUA		22	osa-miR2118g	97	118	3′	139	−71	43.88	1.17	**F003IAL01CL16O** 6
tae-miR2118b	UUCCCGAUGCCUC**U**CAUUCCUA		22	osa-miR2118b	96	117	3′	138	−59	46.38	0.92	**F003IAL01CL16O** 6
tae-miR2118e	UU**U**CUGAUGUCUCCCAUUCCUA		22	zma-miR2118e	98	119	3′	140	−53	42.14	0.90	**F1ADE5F01C34UT** 6
tae-miR2118f	UU**U**CUGAUGCCUCCCAUUCCUA		22	osa-miR2118f	96	117	3′	138	−49	40.58	0.88	**F1ADE5F01C68L2**
tae-miR2118f	UUCCUGAUGCCUCCCAUUCCUA		22	osa-miR2118f	101	122	3′	143	−49	47.55	0.73	F1ADE5F01D1MVB
tae-miR2905	**C**ACAUGUCAGUG**C**CAAAGGCA		21	osa-miR2905	61	81	3′	102	−53	54.90	0.94	F1ADE5F01EPHEM
tae-miR2905	**C**ACAUGUCAGUGAC**C**AAGGCA		21	osa-miR2905	61	81	3′	102	−57	54.90	1.02	F2MIQBM02EQP10
tae-miR5049	**A**CCUAAAUACUUGU**A**GUUGGG		21	hvu-miR5049	20	40	5′	88	−56	38.64	1.65	**F0RUNSI02HO6UW**
tae-miR5050	**G**UGAGGUCGUUCAACC**G**GCAA		21	hvu-miR5050	94	114	3′	135	−74	61.48	0.89	**F1ADE5F01D1QBQ**

1 Where two similar known miRNAs gave equally close matches to a sequence, the evolutionarily closest match is given.

2 Matched sequence reads shown in bold were also predicted to form miRNA hairpins by miRPara.

3 Mismatches to the conserved miRNA sequence are underlined and in bold.

4 MFE  =  Minimum Folding free Energy of predicted hairpin secondary structure.

5 MFEI  =  Minimum Folding Energy Index, calculated as described by Yin et al. [40].

6 miRPara did not predict these hairpins, but predicted a pre-miRNA on the complementary strand. For F003IAL01CL160, which contains two adjacent pre-miRNA hairpins, miRPara predicted the same strand for one but the complementary strand for the other.

In addition, 24 hairpins were found that passed the pre-miRNA criteria, but were marked as lower confidence predictions (Table 3) due to being members of TE-related miRNA families (next section).

**Table 3:** TE-related possible miRNA coding sequences (TE-miR) on chromosome 1AL.

miRNA from chromosome 1AL			Conserved miRNA^1^	Mature miRNA location			Pre-miRNA statistics				Matched sequence read(s)^2^
ID	Sequence^3^	Length(nt)	ID	Start	End	Arm	Length	MFE^4^	GC%	MFEI^5^	ID
tae-miR437	AAAGUUAGAGAAGUUUGACUU	21	osa-miR437	172	192	3′	199	−52	26.13	1.01	F1ADE5F01CDHM0
tae-miR818a	AAU**GU**CUUAUAUUAUGGGACGG	22	osa-miR818a	65	86	3′	107	−62	33.64	1.73	**F2MIQBM01B5CF9, F2MIQBM01BDXG5**
tae-miR1118	**UC**CUACAUUAUGGAAUGGAGGGA	23	tae-miR1118	20	42	5′	106	−46	38.68	1.12	F1NBZEY01AFQVZ
tae-miR1118	CACUACAUU**G**UG**A**AAUGGAGGGA	23	tae-miR1118	201	223	3′	234	−178	46.58	1.63	F1ADE5F01EHKQU
tae-miR1118	CACUACAUU**G**UGGAA**C**GGAGGGA	23	tae-miR1118	192	214	3′	235	−176	46.81	1.60	F1ADE5F01EHKQU
tae-miR1121	AGUAGUGAUCUAAACGCUCUUA	22	tae-miR1121	62	83	3′	104	−58	32.69	1.69	**F1NBZEY01AUMIT**
tae-miR1121	A**A**UAGUGAUCUAAACGCUCUUA	22	tae-miR1121	115	136	3′	157	−64	32.48	1.25	F0RUNSI01D7KCV
tae-miR1121	A**A**UAGUGAUCUAAACGCUCUUA	22	tae-miR1121	115	136	3′	157	−59	33.12	1.13	F0RUNSI01D7KCV
tae-miR1121	A**A**UAGUGAUCUAAACGCUCUUA	22	tae-miR1121	66	87	3′	108	−59	31.48	1.74	**F0RUNSI02GF88P**
tae-miR1121	**UU**UAGUGAUCUAAACGCUCUUA	22	tae-miR1121	64	85	3′	106	−51	29.25	1.64	**F1NBZEY02HSASV**
tae-miR1121	AGUA**U**UGAUCUAAAC**A**CUCUUA	22	tae-miR1121	61	82	3′	103	−40	29.13	1.34	**F2MIQBM01CFLL6**
tae-miR1125	AACCAACGAGACC**G**ACUGCGGCGG	24	tae-miR1125	20	43	5′	126	−96	42.06	1.81	F1ADE5F01EHKQU
tae-miR1125	AACCAACGAGACC**G**ACUGCGGCGG	24	tae-miR1125	20	43	5′	153	−102	39.87	1.68	F0RUNSI01BM4RW
tae-miR1127	AACUACUCCCUCCGUCCCAUA	21	bdi-miR1127	20	40	5′	119	−53	36.13	1.23	F003IAL01CP7OA
tae-miR1127	**U**ACUACUCCCUCCGUCC**U**AUA	21	bdi-miR1127	20	40	5′	114	−50	42.11	1.04	F0RUNSI02IG9O8
tae-miR1128	UACUACUCCCUCCGU**U**CCAAA	21	ssp-miR1128	20	40	5′	94	−27	41.49	0.68	F1ADE5F01DQMRT
tae-miR1128	UACUACUCCCUCCGUCCCA**U**A	21	ssp-miR1128	20	40	5′	100	−48	38.00	1.26	F003IAL01C154D
tae-miR1133	**U**AUAUACUCCCUCCGUCC**C**AAA	22	tae-miR1133	20	41	5′	96	−37	39.58	0.96	F2MIQBM02EYQFE
tae-miR1139	**U**AGUAACAUA**G**ACUAGUAACA	21	bdi-miR1139	20	40	5′	70	−25	40.00	0.91	F2MIQBM01A78VO
tae-miR1139	**U**AGUAACAUA**G**ACUAGUAACA	21	bdi-miR1139	20	40	5′	84	−29	25.00	1.37	F1NBZEY02F39NT
tae-miR1139	**U**AGUAACAUA**G**ACUAGUAACA	21	bdi-miR1139	20	40	5′	90	−39	40.00	1.08	F1ADE5F01E4WAQ, F003IAL01BA096
tae-miR1439	UUUUGGAACGGAG**A**GAGUAU**G**	21	osa-miR1439	62	82	3′	103	−38	38.83	0.95	F0RUNSI02G0499
tae-miR5203	ACUUAUUAUGGA**U**CGGAGGGA	21	bdi-miR5203	83	103	3′	124	−52	42.74	0.98	F0RUNSI01BLKK9
tae-miR5203	ACUUAUUAUGGA**U**CGGAGGGA	21	bdi-miR5203	84	104	3′	125	−44	32.80	1.07	F2MIQBM02DJIDZ

1 Where two similar known miRNAs gave equally close matches to a sequence, the evolutionarily closest match is given.

2 Matched sequence reads shown in bold were also predicted to form miRNA hairpins by miRPara.

3 Mismatches to the conserved miRNA sequence are underlined and in bold.

4 MFE  =  Minimum Folding free Energy of predicted hairpin secondary structure.

5 MFEI  =  Minimum Folding Energy Index, calculated as described by Yin et al. [40].

## Many Wheat-specific miRNAs are Related to DNA Transposons

### Highly represented miRNAs on chromosome 1AL

A large majority of the putative pre-miRNAs found in the 1AL survey sequences belonged to just 19 miRNA families, each of which gave 10 or more hits (Tables 4 and S1). All of these pre-miRNA sequences were screened for similarity to known repeat sequences using RepeatMasker (see Materials and Methods). From the cumulative length of the putative pre-miRNAs, 85.4% matched one or more known repetitive sequence, almost all of which were DNA transposons. The majority of these (62.82% of all sequences) matched Miniature Inverted Terminal repeat Elements (MITEs). 15 of the highly represented miRNAs matched MITEs from the Stowaway family, Mariner sub-family, while 164/168 hits for miR1139 matched a single MITE from the Tourist family, Harbinger sub-family. A further 20.72% of the sequences matched CACTA elements; in most cases, apart from miR1131, these were adjacent to or overlapping a MITE within the same sequence. The only repetitive elements detected that were not DNA transposons were for ath-miR5021, which contains a (GAA)_5_ repeat, so several of the putative hits for this miRNA derived from degenerate trinucleotide repeats. For 9 miRNAs, every copy in the 1AL survey sequences matched a known repetitive element, including the most highly represented (miR1117 & miR1122, with over 400 copies each). For the remaining 10 miRNAs, the majority of occurrences matched known repeats but some did not (see Table 4 for details). Where five or fewer non-repeat sequences were identified for a highly represented miRNA family, these were marked as possible TE-related miRNAs at low confidence (Table 3). In addition, hits for miR437, miR818a and miR1121 were all indicated to be TEs, but as each was only present in a single or few reads, these were also labelled as possible TE-miRNAs. For miR1120 and miR1436, for which 54 and 150 occurrences respectively did not match any known repeats, the large number of these occurrences suggests that they may come from unknown repeat sequences. All the sequences containing transposon-related miRNAs were also used to search the wheat EST database. One instance each of miR1120, miR1136 & miR1139, 4 copies of miR1122, and 5 of miR1135 gave 100% identical matches to an EST, indicating that despite being DNA transposons, some of these sequences can be transcribed from chromosome 1AL.

**Table 4:** Highly represented repeat-related miRNA families in 1AL survey sequences.

Conserved miRNA	Sequence	Hits passinghairpin criteria	Hits matchingknown repeats	Families of knownrepeats matched^1^
tae-miR1117	UAGUACCGGUUCGUGGCACGAACC	471	471	CACTA, Unknown
tae-miR1118	CACUACAUUAUGGAAUGGAGGGA	76	73	Mariner
hvu-miR1120 tae-miR1120	ACAUUCUUAUAUUAUGGGACGGAG ACAUUCUUAUAUUAUGAGACGGAG	220	166	Mariner, CACTA
bdi-miR1122 far-miR1122	UAGAUACAUCCGUAUUUGGA UAGAUACAUCCGUAUCUAGA	437	437	Mariner
tae-miR1125	AACCAACGAGACCAACUGCGGCGG	24	22	Mariner
tae-miR1126	UCCACUAUGGACUACAUACGGAG	72	72	Mariner
bdi-miR1127 tae-miR1127	AACUACUCCCUCCGUCCGAUA UCCUUCCGUUCGGAAUUAC	14	12	Mariner, CACTA
ssp-miR1128 tae-miR1128	UACUACUCCCUCCGUCCCAAA UACUACUCCCUCCGUCCGAAA	99	97	Mariner, CACTA
tae-miR1130	CCUCCGUCUCGUAAUGUAAGACG	66	31	Mariner, CACTA
tae-miR1131	UAGUACCGGUUCGUGGCUAACC	182	182	CACTA
tae-miR1133	CAUAUACUCCCUCCGUCCGAAA	61	60	Mariner
bdi-miR1135 tae-miR1135	UUUCGACAAGUAAUUCCGACCGGA CUGCGACAAGUAAUUCCGAACGGA	201	201	Mariner
tae-miR1136	UUGUCGCAGGUAUGGAUGUAUCUA	226	226	Mariner
tae-miR1137	UAGUACAAAGUUGAGUCAUC	146	146	Mariner
tae-miR1139	AGAGUAACAUACACUAGUAACA	168	164	Harbinger
hvu-miR1436	ACAUUAUGGGACGGAGGGAGU	397	247	Mariner, CACTA
osa-miR1439	UUUUGGAACGGAGUGAGUAUU	172	172	Mariner
ath-miR5021	UGAGAAGAAGAAGAAGAAAA	19	19	Trinucleotide, CACTA
bdi-miR5203	ACUUAUUAUGGACCGGAGGGA	11	9	Mariner

1 Repeats were classified using the system proposed by Wicker et al. [29].

### MITE-related sequences are significantly represented in wheat small RNA libraries

**Figure 1:**
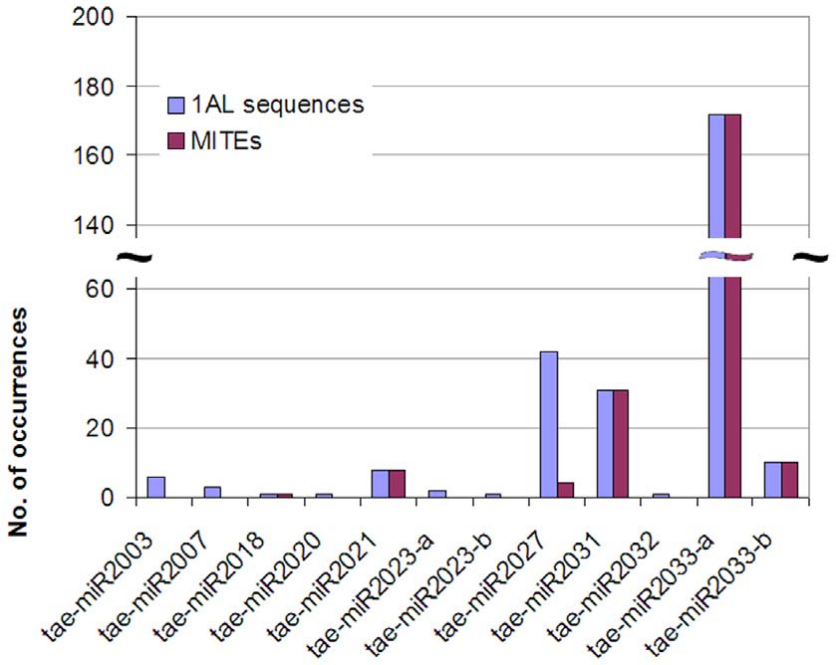
Representation of putative novel wheat miRNAs in 1AL survey sequences. 39 putative novel wheat miRNAs reported by Wei et al. [17] were screened for presence in the 1AL survey sequences. ‘1AL sequences’  =  number of sequences similar to each putative miRNA with good hairpin characteristics. MITEs  =  the number of the same sequences that were identified as Miniature Inverted Terminal repeat Elements.

The majority of these transposon-related miRNAs were all first identified in the same study by high-throughput sequencing of a wheat small RNA library, followed by identification of hairpin precursors in wheat ESTs ([16]; note that miR1117-miR1139 are referred to as TamiR501-TamiR523 in the original study); putative paralogs of some of these have subsequently been identified computationally in other grass species [20], [27], [28]. To investigate whether this approach preferentially detects transposon-related miRNAs in wheat, we carried out the same analysis on 39 putative novel wheat miRNAs that are not currently in miRBase, discovered by Wei et al. [17] using a similar small RNA library sequencing strategy. Of the 39 putative novel wheat miRNAs, 12 were detected in 1 or more copies in the 1AL survey sequences (Figure 1 and Table S1); all matches were screened for the presence of repeats. In all, 6 of the 12 putative novel wheat miRNAs found in chromosome 1AL were not repetitive sequences (Table 5); however, miR2023a & b matched the same sequences previously detected by hvu-miR5050, but in the opposite arms of the hairpin, and were in fact reverse complements of each other with the 2-nt 3′ overhang characteristic of a miRNA:miRNA* duplex. Similarly, miR2032 proved to be identical to miR5200 and matched the same sequence. The remaining 3 non-repetitive sequences (miR2003, miR2007 & miR2020) all of which also were supported by miRNA* sequences [17] are likely to be genuine novel wheat miRNAs. One of the matches to miR2007 also gave a perfect EST match, indicating that this sequence is expressed from chromosome 1AL.

**Table 5:** Putative novel wheat miRNAs discovered by Wei et al. [17] identified in chromosome 1AL survey sequences.

Putative wheat-specific miRNA				Mature miRNA location			Pre-miRNA characteristics				Matched sequence read
ID	Length	Sequence^5^	Mismatch	Start	End	Arm	Length	MFE^6^	GC%	MFEI^7^	
tae-miR2003	22	CGGUUGGGCUGUAUGAUGGCGA	0	73	94	3′	115	−56.6	44.35	1.11	F0RUNSI02FRR0A
tae-miR2003	22	CGGUUGGGCUGUAUGAUGGCGA	0	74	95	3′	116	−60.2	43.10	1.20	F0RUNSI01AQXNL
tae-miR2003	22	CGGUUGGGCUGUAUGAUGGCGA	0	76	97	3′	118	−58.9	42.37	1.18	F1NBZEY01D4XAJ
tae-miR2003	22	CGGUUGGGCUGUAUGAUGGCGA	0	74	95	3′	116	−63.5	43.97	1.25	F1ADE5F01DCEMU
tae-miR2003	22	CGGUUGGGCUGUAUGAUGGCGA	0	74	95	3′	116	−62.8	43.10	1.26	F1ADE5F01A2VHK
tae-miR2003	22	CGGUUGGGCUGUAUGAUGGCGA	0	74	95	3′	116	−62.8	43.10	1.26	F1ADE5F01A9LZ0
tae-miR2007	22	CAAGAUAUUGGGUAUUUCUGUC	0	45	66	3′	87	−35.3	26.44	1.53	F1ADE5F01CRURV
tae-miR2007^1^	22	CAAGAUAUUGGGUAUUUCUGUC	0	46	67	3′	88	−42.2	26.14	1.83	F1ADE5F01BJ39D
tae-miR2007	22	CAAGAUAUUGGGUAUUUCUGUC	0	46	67	3′	88	−40.7	27.27	1.70	F1ADE5F01BJ39D
tae-miR2018^2^	20	GC**U**CGUCUAGCUCAGUUGGU	1	20	39	5′	328	−104	41.77	0.76	F003IAL01AFVSB
tae-miR2020	21	AUAGCAUCAUCCAUCCUACC**C**	1	20	40	5′	109	−53.8	49.54	1.00	F1ADE5F01DWH7W
tae-miR2023-a^3^	22	UUUUGCCGGUUGAACGACCUCA	0	20	41	5′	142	−70.2	62.68	0.79	F1ADE5F01D1QBQ
tae-miR2023-a^3^	22	UUUUGCCGGUUGAACGACCUCA	0	20	41	5′	140	−74.1	62.14	0.85	F1ADE5F01D2FWK
tae-miR2023-b^3^	22	UUUUGCUGGUUGAACGACCUCA	0	20	41	5′	142	−75.1	61.97	0.85	F1ADE5F01D2FWK
tae-miR2032^4^	21	UGUAGAUACUCCCUAAGGCUU	0	76	96	3′	117	−38.8	38.46	0.86	F2MIQBM01ARRO6

1 This pre-miRNA had an identical wheat EST match.

2 pre-miRNA sequence matched a transposable element, but only one copy was found in 1AL.

3 miR2023 and miR5050 (see Tables 1 & 2) derive from opposite arms of the same miRNA:miRNA* duplex.

4 miR2032 is identical to miR5200 (see Table 1).

5 Mismatches to the conserved miRNA sequence are underlined and in bold.

6 MFE  =  Minimum Folding free Energy of predicted hairpin secondary structure.

7 MFEI  =  Minimum Folding Energy Index, calculated as described by Yin et al. [40].

Of sequences matching the putative novel wheat miRNAs, 55.99% of the sequences also matched known repetitive elements, all of which were MITEs or unclassified repeats. As before, all putative miRNAs that gave 10 or more matches were found to be transposon-related, and for 5 of these putative miRNAs every occurrence was marked as a transposon (Fig. 1). miR2018 only had 1 match in the 1AL survey sequences and also matched a MITE, while miR2027 was exceptional in that it was present in 42 sequences but only 4 of these matched any known repeat. As with some of the highly repeated miRNAs examined above, perfect EST matches were found for some occurrences of miR2027 and miR2031, suggesting that these are also transcribed from chromosome 1AL. The substantial presence of MITE-related sequences in small RNA libraries prepared in 2 different laboratories suggests that these make up a significant component of the small RNA population in wheat cells.

## Prediction of Putative miRNA Sequences on Chromosome 1AL by Hairpin Structure

### Comparison of similarity search and predictive methods for miRNA annotation

**Figure 2:**
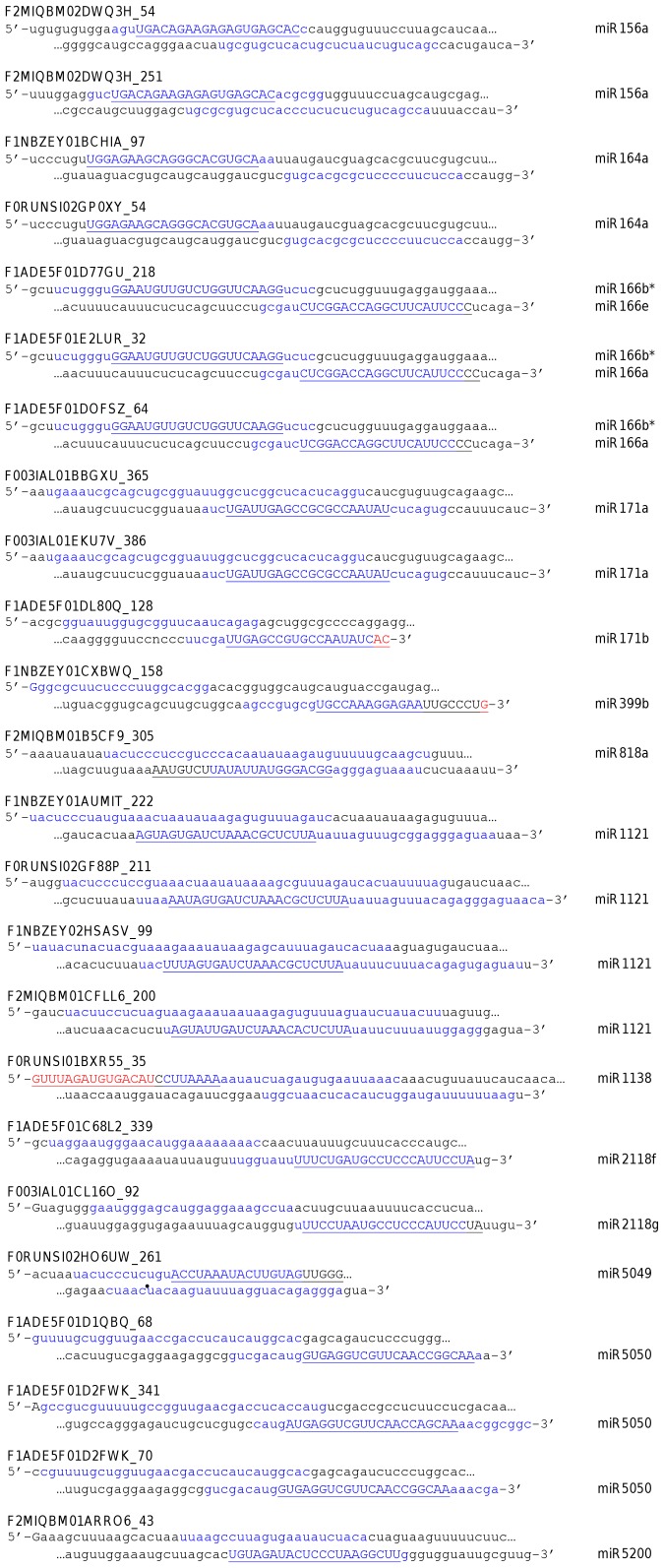
Comparison of mature miRNA locations in hairpins predicted by sequence similarity and by miRPara. Each sequence is preceded by the unique sequence read ID with the start position of the predicted hairpin appended. The ranges of possible mature miRNA locations predicted by miRPara are shaded in blue. The location of the conserved mature miRNAs found by similarity search is shown in capital letters and underlined. Nucleotides of the conserved mature miRNA that are found outside the predicted hairpin are highlighted in red.

Searching on the basis of known miRNAs is an effective means of locating conserved miRNA genes, although novel miRNA sequences cannot be identified by this method. Therefore we also used an SVM-based algorithm to predict putative miRNA hairpins in the 1AL survey sequences. Of the several programs available, we selected miRPara [24]. A plant miRNA-trained version of this algorithm is available, and it uses UNAFold to predict RNA secondary structure, making it comparable to our similarity-based procedure. miRPara was used at its highest sensitivity (level 1) to search all the 1AL survey sequences; in total 85,820 possible miRNA hairpins were detected (an average of 1 hairpin for every 25 sequence reads). Owing to the large size of the dataset and the need to produce a secondary structure for every possible sequence, this required a significant amount of computing time. At this point, the predicted miRNA hairpins were compared with those identified using the similarity search method. From the 46 pre-miRNA hairpins predicted with high confidence from similarity searches, 24 were also predicted to be pre-miRNAs by miRPara, while in two more cases (tae-miR2118b & e) miRPara predicted that the complementary strand but not the sense strand of the same sequence read could form a miRNA hairpin. For each hairpin, miRPara predicts a range of possible locations for the mature miRNA sequence; for the hairpins that were identified by both methods, the locations of the mature miRNAs are compared in Figure 2. Generally, all mature miRNA sequences identified by similarity searches overlapped with the range predicted by miRPara for each hairpin, and usually fell entirely within it, although in 4 cases 5 or more nucleotides of the mature miRNAs were outside the predicted range, and for 1 of these (miR1138) more than half the mature miRNA sequence fell outside the hairpin altogether.

### Repetitive elements and candidate novel miRNA candidates in predicted hairpins

**Figure 3:**
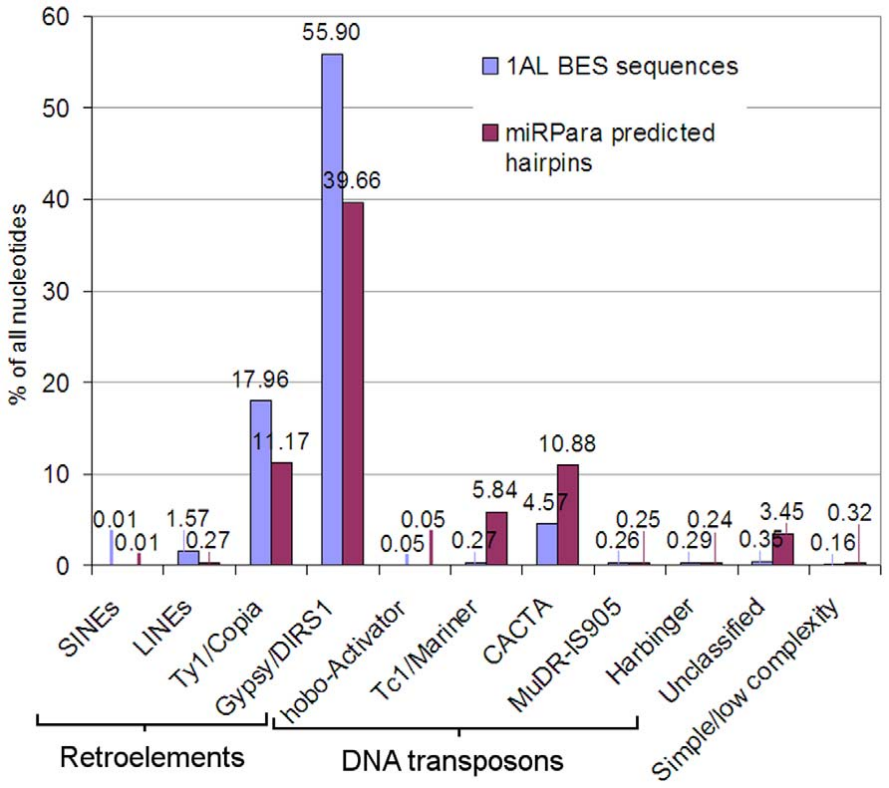
Representation of different repeat element families in BAC-end sequences and predicted hairpins from 1AL. 1AL BES sequences were obtained as described previously [26]. Hairpins were predicted using miRPara’s plant miRNA prediction model. Repeat content was calculated as the cumulative length of all nucleotides marked as being part of repetitive elements, and expressed as a percentage of the total length of each dataset. The 1AL BESs included 7,568,093 nt of which 81.97% matched known repeat elements. The predicted hairpins included 8,081,278 nt of which 72.8% matched known repeat elements.

All the hairpins predicted by miRPara were then screened for repeat element content; by cumulative nucleotide length, 72.8% of the hairpin sequences matched known repeats. The cumulative length of all the putative hairpins was 8.08 MB, a similar sized sample of chromosome to that previously obtained by BAC-end sequencing [26], which totalled 7.57 MB. Therefore, the proportions of different repeat classes detected in predicted hairpins were compared with those found in BAC-end sequences (Figure 3). All the same repeat classes were represented in both datasets, with the relatively uncommon repeats occurring at similar frequencies. However, the predicted hairpins showed a relatively reduced proportion of all the retroelements, and an enrichment for DNA transposons, especially Mariner elements which were 22-fold more abundant in the hairpin sequences. All of the repeat elements were masked, and the sequences then compared with all wheat EST sequences using BLAST, to identify expressed sequences. Only positive strand hits with 0 or 1 mismatch to the predicted hairpin were retained. These were then further screened against all Triticeae ESTs and the *Brachypodium distachyon* genome sequence, and any hits to sequences annotated as encoding tRNA, rRNA or proteins were eliminated. After this, 59 sequences remained, which can be considered as potentially encoding novel miRNAs. The highest probability miRNA prediction for each of these hairpins is shown in Table S2. However, further experimental work and comparison with wheat small RNA datasets is required to confirm which of these hairpins is cleaved to produce a viable mature miRNA sequence.

## Discussion

As genomic sequences of crop species become available, characterizing their miRNA populations is an important element in developing a full picture of their gene expression and regulation. However, the complex small RNA population and repeat-rich genome of crops such as wheat necessitates a cautious approach to miRNA annotation [25]. In this study, we developed 2 scripts, SUmirFind and SUmirFold, that together provide a straightforward and rapid method for identifying putative conserved miRNAs in any BLAST database (Methods S1). Using these scripts, we carried out the first identification of miRNA coding sequences in *T. aestivum* L. var. Chinese Spring, chromosome 1AL. Previous studies of miRNAs in bread wheat have been based on sequencing of small RNA libraries and/or similarity searches within wheat EST sequences [16]–[19]. Searching for miRNAs in genome or chromosome survey sequences has the advantage that miRNAs that are only expressed under specific conditions, and so may not be represented in small RNA libraries or EST sequences, can also be detected. In this study, 42 different putative miRNA coding sequences were identified with high confidence in chromosome 1AL, representing 14 conserved miRNA families. Of these miR437, miR2118, miR2905, miR5049, miR5050, miR5075 & miR5200 have not previously been reported from wheat EST sequences, while miR399k is also a new representative of the miR399 family. The downside of searching genome survey sequences is that miRNA-like sequences may be silent or pseudo-miRNAs. We were able to find 98% identical EST matches (in practice, 0 or 1 mismatch to the pre-miRNA sequence) to 8 putative miRNA coding sequences, including the last 3 newly detected families above. The presence of an identical EST is a strong indicator that these particular pre-miRNA sequences are expressed, rather than orthologous loci on other chromosomes; 1 mismatch is permitted because of the possibility of sequencing errors and point mutations between different cultivars. This is a fairly stringent requirement, and the other 34 putative miRNA coding sequences may also be expressed but not currently represented by near-identical ESTs.

In addition, a further 22 conserved miRNA families were identified with much higher copy numbers (from 10 to over 400 copies) than might be expected for a typical miRNA. Repeat analysis demonstrated that all of these families are related to repeat sequences, and the great majority to MITEs, most commonly of the Mariner sub-family. These are small (50–500 bp) DNA transposons that feature terminal inverted repeats (TIRs) of 10–30 bp [29]; thus they can have a similar size and secondary structure to pre-miRNAs, with the TIRs forming the hairpin stem. Other repeat elements detected were Harbinger-type MITEs and CACTA elements. In addition, most hits for ath-miR5021 corresponded to degenerate trinucleotide repeats and so could not be considered as miRNAs.

Recently it has been proposed that some miRNAs evolved from transposable elements (specifically MITEs) by read-through transcription of their terminal repeats [30], and TEs that encode miRNAs and siRNAs have been reported in rice and *Arabidopsis thaliana* [31]. Given the high repeat content of the *T.aestivum* genome, it might be expected to contain TE-related miRNAs. However, distinguishing which of these TE-related sequences really function as miRNAs is far from straightforward. In rice, 91 TE-related miRNA sequences have been analyzed in detail, and only 9 were found to satisfy current miRNA annotation criteria [32]. However, viable gene targets and evidence for miRNA-like site-specific cleavage of these targets were found for some of the other TE-related miRNAs as well. Some of these TE-related miRNAs were also found on chromosome 1AL, including miR437 and members of the MIR818 gene family (miR818a, miR1436 & miR1439). Low genomic copy number is one characteristic of bona fide TE-derived miRNAs, so the single copies of miR437 and miR818a found on 1AL may well represent genuine miRNAs (Table 3). However, the high copy number miRNA families identified here cannot be annotated as miRNA genes; individual cases that do not match known repeats were marked as possible miRNAs, but at low confidence as they could also correspond to unknown repeats. Furthermore, we compared pre-miRNA-like sequences on 1AL from two different sets of putative novel wheat miRNAs generated from independent wheat small RNA libraries: tae-miR1117–1139 [16] and tae-miR2001–2033 [17]. From these miR1138, miR2003, miR2007 & miR2020 were not repeat related and therefore are probable bona fide novel miRNAs on chromosome 1AL, while miR2023 & miR2032 were identical to hvu-miR5050* and bdi-miR5200 respectively. The majority of the remaining matches to both datasets were TE-related, indicating that TE sequences are significantly represented in the small RNA population in wheat cells; perfect EST matches to instances of miR1120, miR1122, miR1135, miR1136, miR1139, miR2027 & miR2031 indicate that some of these sequences are also transcribed from chromosome 1AL. Surprisingly, however, none of the same TE-related miRNAs were identified in both small RNA library studies, which raises the possibility that some could be artifacts produced from, for example, degradation of silent copies of common TEs within gene introns and untranslated regions. Alternatively, they may be wheat TEs in the process of evolving into miRNA genes. Further functional analysis is required to determine which of these high copy number putative miRNAs is biologically significant.

As homology based searching is limited to identifying conserved miRNAs, we also tested a SVM-based predictive algorithm to identify miRNAs in the 1AL survey sequences. This showed that identifying wheat miRNAs from predicted secondary structure alone is very difficult, as over 8 MB (1.5% of the predicted size of the chromosome) of sequences were able to form miRNA-like hairpins. Compared with the more random sample of the chromosome generated by BAC-end sequencing [26], predicted hairpin-forming sequences were enriched for DNA transposons, especially MITEs, again indicating that they can easily be mistaken for miRNAs; however, these still only comprised about 17% of all hairpins (Figure 3), with retroelements making up over 50% and non-repetitive sequences 27.8%. There was only average overlap between the hairpins predicted by the SVM and these identified through homology searches. This may be because the miRPara plant model was trained on an older release of miRBase, version 13.0 [24] which contained far fewer grass miRNA sequences than are currently available; re-training with miRNA set more weighted towards grasses or even Triticeae might give better results. However, even then it may be difficult to distinguish recently-evolved TE-related miRNAs from TE sequences, as there may be very little difference in their secondary structures. Taking this into account, using miRPara we were able to identify 59 unique 1AL sequences that form hairpins, are expressed, and do not correspond to any known repeat, miRNA or protein coding sequence. Future work will reveal which of these can be cleaved to produce functional miRNAs.

Our data from chromosome 1AL show broad similarity in the quantity and variety of miRNA coding sequences to results recently obtained from survey sequencing of *T. aestivum* chromosome 5A [5] and 4A [33], although the selection of miRNAs varies from chromosome arm to chromosome arm. It is notable that the only miRNAs found on all 5 chromosome arms are those shown here to be TE-related. Given the difficulty of differentiating between TE-related miRNAs and miRNA-like TEs, and the possibility of the same miRNA loci being found on different chromosomes but not all being expressed, we propose the following 3-tier strategy to annotating miRNAs in grass genomes: i) sequences that pass the miRNA identification criteria and for which there is evidence of transcription (e.g. corroborating EST) can be annotated as miRNAs; ii) sequences that pass the miRNA identification criteria and are not repeat-related, but lack evidence of expression, can be annotated as ‘hypothetical miRNAs’; iii) all putative miRNAs found in wheat genomic sequences that are similar to TE sequences, and/or have a copy number higher than 10, should be annotated as TE-miRNAs and only regarded as tentative predictions until they can be confirmed with functional data. This is a simple and easily applicable strategy, which if adopted should avoid later confusion and the need to re-annotate large numbers of sequences incorrectly labelled as miRNAs.

## Materials and Methods

### Reference miRNA and Wheat Chromosome 1AL Sequences

For computational identification of conserved miRNAs with putative homologs on chromosome 1AL, previously identified plant mature miRNA and pre-miRNA sequences were downloaded from miRBase release 17 (April 2011; [34]), containing 3362 miRNAs from 46 different plant species. Where multiple mature miRNAs had identical sequences, only one was retained; moreover in accordance with the criteria for miRNA annotation [8], a small number of miRNA families for which there is currently no experimental confirmation in any species were also removed from the list, leaving 2043 miRNA sequences from 897 families.

Seeds of *T.aestivum* L. (cv Chinese Spring) chromosome 1A double ditelosomic line were provided by Bikram S. Gill (Kansas State University, Manhattan, KS). Aqueous suspensions of mitotic chromosomes were prepared from root tips, stained with 2 µg/ml 4′,6-diamidino-2-phenylindole, and sorted using a FACSVantage SE flow cytometer (Beckton Dickinson) as previously described [3]. Prior to sequencing, the DNA of the chromosome arm was amplified by using the Illustra GenomiPhi V2 DNA amplification kit (GE Healthcare Bio-sciences) in a 20 µl reaction volume with the method described by Šimková et al. [35]. Using 5 µg of amplified DNA, the 454 sequencing library was prepared, processed and sequenced with the GS Titanium General Library Preparation Kit, the GS FLX Titanium LV emPCR (Lib-L) Kit, and the GS FLX Titanium Sequencing (XLR70) Kit (all Roche Diagnostics) following the manufacturer’s instructions.

### Identifying Conserved miRNAs by Similarity and Secondary Structure

After trimming the 454 sequence reads to remove low quality sequence, BLAST databases were constructed from the sequences using the BLAST+ stand-alone toolkit, version 2.2.24, from the NCBI [36]. Conserved miRNAs were identified using two newly written Perl scripts, SUmirFind and SUmirFold (see Methods S1). SUmirFind uses blastn with parameters optimized for short sequences, and to give longer hits with mismatches the same score as shorter hits without mismatches (-task blastn-short -ungapped -penalty -1 -reward 1). It then filters the hits, eliminating any with >2 bases different from the miRNA query, and gives output in the format of a standard BLAST results table (output format 6).

SUmirFold then uses the output of SUmirFind (or any other BLAST results table in the same format) to search for viable pre-miRNA sequences. The sequence in which the hit was found is first retrieved from the BLAST database, converted to RNA and reverse complemented if necessary, and then its secondary structure predicted using UNAFold version 3.8 [37] which is an implementation of the Zuker algorithm for single-stranded RNA structure prediction. The lowest minimum free energy (MFE) structure is examined for base-pairing within the putative mature miRNA sequence, and eliminated if it fails to meet the specified criteria (see results section). For all hits passing the criteria, the part of the sequence containing the putative miRNA and its surrounding hairpin (defined arbitrarily as starting and finishing 20 nt further away from the head of the stem-loop than the outer end of the miRNA:miRNA* duplex) is excised, re-folded, and tested to see whether it has the characteristics of a pre-miRNA structure. Output is given in the form of a results table, as well as fasta files and structure diagrams of the positive hairpins.

### Prediction of miRNAs in Genomic Sequences

Prediction of possible miRNA coding sequences was carried out using miRPara release 4.1 [24], an SVM-based algorithm trained against a set of 1215 plant miRNAs from miRBase release 13.0. As with SUMirFold, miRPara utilizes UNAFold for the prediction of RNA secondary structure. For screening of 1AL 454 sequences, miRPara was used with default settings (apart from specifying the model for plant miRNAs).

### Identifying Repetitive Elements

A semi-automated pipeline was used to identify and mask repetitive elements from the 1AL survey sequences, using RepeatMasker version 3.2.9 (www.repeatmasker.org) with CrossMatch (www.phrap.org/phredphrapconsed.html) as alignment algorithm. First of all, three consecutive runs of RepeatMasker were carried out using default settings with 2 different custom libraries in the following order: TREP release 10 (http://147.49.50.65/ITMI/Repeats/), and a merged library of Repbase Update [38], and TIGR plant repeats [39]. Sequences matching known repeats were masked with an ‘N’.

## Supporting Information

Table S1 **Putative conserved miRNA coding sequences from chromosome 1AL.** Full details of all putative miRNAs identified by conservation, including pre-miRNA hairpin sequences.
(XLS)

Table S2 **Candidate novel miRNA coding sequences from chromosome 1AL.** Details of miRPara-predicted hairpins that showed no similarity to known miRNAs, proteins, repeats or other RNA species, but for which there is evidence of expression.
(XLS)

Methods S1 **SUmirFind & SUmirFold.** Perl scripts for identification of miRNAs by conservation.
(PDF)

## References

[1] Gill BS, Appels R, Botha-Oberholster AM, Buell CR, Bennetzen JL (2004). A workshop report on wheat genome sequencing: international genome research on wheat consortium.. Genetics.

[2] Feldman M, Lupton FGH, Miller TE, Smartt J, Simmonds NW (1995). Wheat..

[3] Kubaláková M, Vrána J, Cíhalíková J, Šimková H, Doležel J (2002). Flow karyotyping and chromosome sorting in bread wheat (*Triticum aestivum* L.).. Theor Appl Genet.

[4] Wicker T, Mayer KF, Gundlach H, Martis M, Steuernagel B (2011). Frequent Gene Movement and Pseudogene Evolution Is Common to the Large and Complex Genomes of Wheat, Barley, and Their Relatives.. Plant Cell.

[5] Vitulo N, Albiero A, Forcato C, Campagna D, Dal Pero F (2011). First Survey of the Wheat Chromosome 5A Composition through a Next Generation Sequencing Approach.. PloS ONE.

[6] Jones-Rhoades MW, Bartel DP, Bartel B (2006). MicroRNAs and their regulatory roles in plants.. Annu Rev Plant Biol.

[7] Voinnet O (2009). Origin, biogenesis and activity of plant microRNAs.. Cell.

[8] Meyers BC, Axtell MJ, Bartel B, Bartel DP, Baulcombe D (2008). Criteria for Annotation of Plant microRNAs.. Plant Cell.

[9] Bartel DP (2004). MicroRNAs: genomics, biogenesis, mechanism and function.. Cell.

[10] Yang J-S, Phillips MD, Betel D, Mu P, Ventura A (2011). Widespread regulatory activity of vertebrate microRNA* species.. RNA.

[11] Llave C, Kasschau KD, Rector MA, Carrington JC (2002). Endogenous and silencing-associated small RNAs in plants.. Plant Cell.

[12] Dugas DV, Bartel B (2004). MicroRNA regulation of gene expression in plants.. Curr Opin Plant Biol.

[13] Khraiwesh B, Zhu J-K, Zhu J (2011). Role of miRNAs and siRNAs in biotic and abiotic stress responses of plants.. Biophys Biochim Acta 10.1016/j.bbagrm.2011.05.001.

[14] Unver T, Namuth-Covert DM, Budak H (2009). Review of Current Methodological Approaches for Characterizing MicroRNAs in Plants.. Int J Plant Genomics 10.1155/2009/262463.

[15] Pantaleo V (2011). Plant RNA silencing in viral defence.. Adv Exp Med Biol.

[16] Yao Y, Guo G, Ni Z, Sunkar R, Du J (2007). Cloning and characterization of microRNAs from wheat (*Triticum aestivum* L.).. Genome Biol.

[17] Wei B, Cai T, Zhang R, Li A, Huo N (2009). Novel microRNAs uncovered by deep sequencing of small RNA transcriptomes in bread wheat (*Triticum aestivum* L.) and *Brachypodium distachyon* (L) Beauv.. Funct Integr Genomics.

[18] Xin M, Wang Y, Yao Y, Xie C, Peng H (2010). Diverse set of microRNAs are responsive to powdery mildew infection and heat stress in wheat (*Triticum aestivum* L.).. BMC Plant Biol.

[19] Dryanova A, Zakharov A, Gulick PJ (2008). Data mining for miRNAs and their targets in the Triticeae.. Genome.

[20] Unver T, Budak H (2009). Conserved microRNAs and their targets in the model grass species *Brachypodium distachyon*.. Planta.

[21] Kantar M, Lucas SJ, Budak H (2011). miRNA expression patterns of *Triticum dicoccoides* in response to shock drought stress.. Planta.

[22] Budak H, Akpinar A (2011). Dehydration Stress-Responsive miRNA in *Brachypodium distachyon:* Evident by Genome-Wide Screening of microRNAs Expression.. OMICS.

[23] Xuan P, Guo M, Liu X, Huang Y, Li W (2011). PlantMiRNAPred: efficient classification of real and pseudo plant pre-miRNAs.. Bioinformatics.

[24] Wu Y, Wei B, Liu H, Li T, Rayner S (2011). MiRPara: a SVM-based software tool for prediction of most probable microRNA coding regions in genome scale sequences.. BMC Bioinformatics.

[25] Jones-Rhoades MW, Meyers BC, Green PJ (2010). Prediction of Plant miRNA Genes..

[26] Lucas SJ, Šimková H, Safár J, Jurman I, Cattonaro F (2011). Functional features of a single chromosome arm in wheat (1AL) determined from its structure.. Funct Integr Genomics 10.1007/s10142–011–0250–3.

[27] Kantar M, Unver T, Budak H (2010). Regulation of barley miRNAs upon dehydration stress correlated with target gene expression.. Funct Integr Genomics.

[28] Unver T, Bakar M, Shearman RC, Budak H (2010). Genome-wide profiling and analysis of *Festuca arundinacea* miRNAs and transcriptomes in response to foliar glyphosate application.. Mol Genet Genomics.

[29] Wicker T, Sabot F, Hua-Van A, Bennetzen JL, Capy P (2007). A unified classification system for eukaryotic transposable elements.. Nat Rev Genet.

[30] Piriyapongsa J, Mariño-Ramírez L, Jordan IK (2007). Origin and evolution of human microRNAs from transposable elements.. Genetics.

[31] Piriyapongsa J, Jordan IK (2008). Dual coding of siRNAs and miRNAs by plant transposable elements.. RNA.

[32] Li Y, Li C, Xia J, Jin Y (2011). Domestication of Transposable Elements into MicroRNA Genes in Plants.. PLoS ONE.

[33] Kantar M, Akpınar BA, Valárik M, Lucas SJ, Doležel J (2012). Chromosome-specific microRNAs in polyploid wheat. Funct Integr Genomics.. In press.

[34] Kozomara A, Griffiths-Jones S (2011). miRBase: integrating microRNA annotation and deep-sequencing data.. Nucleic Acids Res.

[35] Šimková H, Svensson JT, Condamine P, Hribová E, Suchánková P (2008). Coupling amplified DNA from flow-sorted chromosomes to high-density SNP mapping in barley.. BMC Genomics.

[36] Camacho C, Coulouris G, Avagyan V, Ma N, Papadopoulos J (2009). BLAST+: architecture and applications.. BMC Bioinformatics.

[37] Markham NR, Zuker M, Keith JM (2008). UNAFold: software for nucleic acid folding and hybridization..

[38] Jurka J, Kapitonov VV, Pavlicek A, Klonowski P, Kohany O (2005). Repbase Update, a database of eukaryotic repetitive elements.. Cytogenet Genome Res.

[39] Ouyang S, Buell CR (2004). The TIGR Plant Repeat Databases: a collective resource for the identification of repetitive sequences in plants.. Nucleic Acids Res.

[40] Yin Z, Li C, Han X, Shen F (2008). Identification of conserved microRNAs and their target genes in tomato (*Lycopersicum esculentum).*. Gene.

