# Supplementary material for: Sorting the Wheat from the Chaff: Identifying miRNAs in Genomic Survey Sequences of Triticum aestivum Chromosome 1AL
Source: PLoS One. 2012 Jul 17;7(7):e40859. doi: 10.1371/journal.pone.0040859 (PMC3398953; doi:10.1371/journal.pone.0040859)
Supplement: Methods S1 — SUmirFind & SUmirFold. Perl scripts for identification of miRNAs by conservation. (PDF) [file pone.0040859.s003.pdf]

SUmirFind.pl & SUmirFold.pl – Perl scripts for miRNA identification by conservation

Copyright © Stuart J. Lucas, 2012

Digital copies of the scripts and a usage guide are freely available from the author (e-mail: slucas@sabanciuniv.edu) These scripts are written to run on Linux operating systems, but should be relatively easy to adapt for Windows. Perl, BLAST+ and UNAFold software are required to be installed on your computer to use these scripts.

```
#!/usr/bin/perl -w
# SUmirFind.pl - a script that uses ncbiBLAST to search for potential homologs of
known miRNAs

my ( $mirnaquery, $blastdatabase ) = @ARGV or die "Please specify a fasta file
containing the miRNA sequences you wish to search with, and the BLAST database you
wish to search, with full path if it is not in the current directory";

# Convert fasta file into a table of miRNAs

open ( MIRNAS, $mirnaquery ) or die "Could not open $mirnaquery.  Is it in the
right folder?";
open ( MIRNATABLE, ">".$mirnaquery.".tbl" ) or die "Could not open an output
file!";
print MIRNATABLE "# miRNA ID\tsequence";

while($line = <MIRNAS>)
{
        chomp $line;
        if ($line =~ /^>(\S*)\s/)
        {
                print MIRNATABLE "\n$1";
                print MIRNATABLE "\t";
        }
        else
        {
                print MIRNATABLE "$line";
        }
}
print MIRNATABLE "\n";

close MIRNAS;
close MIRNATABLE;

# Populate hash table of miRNAs from newly generated table

my (%QuerymiRNAs);
open ( MIRNADATA, $mirnaquery.".tbl" ) or die "The miRNA table was not generated";
while ($line = <MIRNADATA>)
{
        chomp $line;
        if ( $line =~ /^#/ )
        {
                next;
        }
        elsif ( $line =~ /(\S*)\t(\S*)/ )
        {
                my $idkey = $1;
                my $seqval = $2;
```

```
                $QuerymiRNAs{"$idkey"} = "$seqval";
        }
}

close MIRNADATA;

print "miRNA data analysed....  Now running BLAST for all the miRNAs.  This could
take some time.\n";

# Run BLAST for the specified miRNAs

system ("blastn -task blastn-short -query $mirnaquery -db $blastdatabase -ungapped
-penalty -1 -reward 1 -outfmt 6 -out $mirnaquery.allhits" );

# Filter blast hit table (in output format 6) to remove alignments with >2
mismatches

open ( BLASTHITS, $mirnaquery.".allhits" ) or die "Couldn't find the BLAST
output!";
open ( FILTEREDHITS, ">".$mirnaquery.".results.tbl" ) or die "Results file
failure";

print FILTEREDHITS "# Query ID\tSubject
ID\t%\tlength\tmism\tgaps\tqstart\tqend\tsstart\tsend\tevalue\tbitscore\n";

my $count = 0;
while ( $line = <BLASTHITS> )
{
        chomp $line;
        my ( $qid, $sid, $percent, $allength, $mismatch, $gaps, $qstart, $qend,
$sstart, $send, $evalue, $bitscore ) = split /\t/, $line;
        my $qlength = length $QuerymiRNAs{ $qid };
        my $difference = $qlength - $allength;
        if ( $mismatch + $difference > 2 )
        {
                $count++;
                next;
        }
        else
        {
                print FILTEREDHITS "$line\n";
        }
}
close BLASTHITS;
close FILTEREDHITS;

print "Filtering complete.  $count hits were rejected due to being too short, or
having too many mismatches.\n";
```

```
#!/usr/bin/perl -w
# SUmirFold.pl - a script that, using a BLAST results table, retrieves sequences
from the BLAST database and obtains their predicted secondary structure using
UNAfold, after which viable hairpins are detected and retrieved.

my $goodcount = 0;
my $badcount = 0;
my $suspectcount = 0;
my $loopno = 0;

if ( $#ARGV != 2 )
{
	print "Correct usage is:\n";
	print "perl mirnafold.pl [miRNA query file] [HitTable] [BLASTdb]\n";
}

my ( $mirnaquery, $infile, $blastdb ) = @ARGV or die "Please specify names of a
file containing known miRNAs (in fasta format), BLAST hit table (generated using
'-outfmt 6' when running BLAST) and blast database from which the hit table was
generated.\n";


# Convert fasta file into a table of miRNAs

open ( MIRNAS, $mirnaquery ) or die "Could not open $mirnaquery.  Is it in the
right folder?";
open ( MIRNATABLE, ">".$mirnaquery.".tbl" ) or die "Could not open an output
file!";

print MIRNATABLE "# miRNA ID\tsequence";

while ($line = <MIRNAS>)
{
	chomp $line;
	if ($line =~ /^>(\S*)\s/)
	{
		print MIRNATABLE "\n$1";
		print MIRNATABLE "\t";
	}
	else
	{
		print MIRNATABLE "$line";
	}
}
print MIRNATABLE "\n";

close MIRNAS;
close MIRNATABLE;

# Populate hash table of miRNAs from newly generated table

my (%QuerymiRNAs);
open ( MIRNADATA, $mirnaquery.".tbl" ) or die "The miRNA table was not generated";
while ($line = <MIRNADATA>)
{
	chomp $line;
	if ( $line =~ /^#/ )
	{
		next;
	}
	elsif ( $line =~ /(\S*)\t(\S*)/ )
```

```
        {
                my $idkey = $1;
                my $seqval = $2;
                $QuerymiRNAs{"$idkey"} = "$seqval";
        }
}
close MIRNADATA;
print "miRNA data analysed.  Preparing to fold RNA sequences.\n";

unless ( -e $infile && -f $infile && -r $infile )
{
        die "$infile cannot be accessed.  Does it exist?\n";
}

open ( IN, $infile ) or die "I don't have permission to open $infile!\n";
open ( OUT, ">".$infile.".seqtable" ) or die "Can't open an output file!\n";
open ( OUTTABLE, ">".$infile.".tbl") or die "Can't open an output file!\n";
open ( DISCARD, ">".$infile.".rejects" ) or die "Can't open an output file!\n";
open ( SUSPECT, ">".$infile.".suspect.tbl" ) or die "Can't open an output
file!\n";
open ( SUSPECTOUT, ">".$infile.".suspect.seqtable" ) or die "Can't open an output
file!\n";
open ( LOGFILE, ">".$infile.".log" ) or die "Can't open an output file!\n";
print DISCARD "Matches rejected:\n";
print DISCARD "Seq ID\tmiRNA\tStart\tEnd\tReason for rejection\n";
print OUTTABLE "#Structure       \tMatched    \tConserved\tMatch\tMature
miRNA\tComplement\n";
print OUTTABLE "#Filename        \tSequence ID\tmiRNA
\tlength\tstart\tend\tstart\tend\n";
print SUSPECT "#Structure       \tMatched    \tConserved\tMatch\tMature
miRNA\tComplement\n";
print SUSPECT "#Filename        \tSequence ID\tmiRNA
\tlength\tstart\tend\tstart\tend\n";
system ( "mkdir $infile.initialfolds" );
system ( "mkdir $infile.hairpins" );

while ($line = <IN>)
{
        chomp $line;
        next if $line =~ /^#/;
        next if $line eq "";
        print ".\n";

# Get the values from the table, retrieve the sequence from the BLAST database and
write it to a fasta file

        my ( $qid, $sid, $percent, $allength, $mismatch, $gaps, $qstart, $qend,
$sstart, $send, $evalue, $bitscore ) = split /\t/, $line;
        my $qlength = length $QuerymiRNAs{ $qid };
        $loopno++;
        my $uniqueid = $loopno."_".$sid;
        system ("blastdbcmd -db $blastdb -entry $sid -outfmt %f -out
$uniqueid.fsa" );
        print LOGFILE "Testing hit for $qid on sequence $uniqueid\n";

# Reverse complement the sequence if it is on the negative strand; otherwise just
convert Ts to Us
        if ( $sstart > $send )
        {
                print LOGFILE "This hit is on the negative strand.  Initiating
reverse complement.\n";
```

```
                my $tempseq = "";
                my $defline = "";
                open ( NEGSTRAND, $uniqueid.".fsa" );
                while ( $line = <NEGSTRAND> )
                {
                        chomp $line;
                        if ( $line =~ m/^>/)
                        {
                                $defline = $line;
                        }
                        else
                        {
                                $tempseq = $tempseq.$line;
                        }
                }
                close NEGSTRAND;
                $tempseq = scalar reverse ("$tempseq");
                $tempseq =~ tr/[UT]/a/;
                $tempseq =~ tr/A/u/;
                $tempseq =~ tr/C/g/;
                $tempseq =~ tr/G/c/;
                $tempseq =~ tr/acgu/ACGU/;
                open  ( POSSTRAND, ">".$uniqueid.".fsa" );
                print POSSTRAND "$defline\n$tempseq\n";
                close POSSTRAND;
                $seqlength = length ($tempseq);
                $sstart = 1 + $seqlength - $sstart;
                $send = 1 + $seqlength - $send;
                print LOGFILE "Reverse complement stats (id, length, hit start and
end): $sid, $seqlength, $sstart, $send\n";
        }
        else
        {
                my $tempseq = "";
                my $defline = "";
                open ( DNASTRAND, $uniqueid.".fsa" );
                while ( $line = <DNASTRAND> )
                {
                        chomp $line;
                        if ( $line =~ m/^>/)
                        {
                                $defline = $line;
                        }
                        else
                        {
                                $tempseq = $tempseq.$line;
                        }
                }
                close DNASTRAND;
                $tempseq =~ tr/T/U/;
                open  ( RNASTRAND, ">".$uniqueid.".fsa" );
                print RNASTRAND "$defline\n$tempseq\n";
                close RNASTRAND;
        }

# Adjust ends of putative mature miRNA if it is shorter than query miRNA, and note
'extended' miRNA duplex end
        $sstart = $sstart - $qstart + 1;
        $send = $send + $qlength - $qend;
        my $exstart = $sstart - 2;
```

```
# Run UNAfold on the fasta file
	print LOGFILE "Running UNAfold on $sid\n";
	system ("perl /usr/bin/UNAFold.pl -X 1 --ann ss-count $uniqueid.fsa");

# Examine the Ct file for unpaired bases in the miRNA sequence
	open ( FOLD, $uniqueid.".fsa.ct" ) or die "where is the ct file?";
	my $linecount = 0;
	my $ssflag = 0;
	my $revstart = 0;
	my $revstartbackup = 0;
	my $revend = 0;
	my $revendbackup = 0;
	my $revlength = 0;
	while ($line = <FOLD>)
	{
		chomp $line;
		next if $line =~ /dG/;
		$linecount++;
		next if $linecount < ($exstart);
		next if $linecount > ($send);
		if ( $line =~ /\d*\t\w\t\d*\t\d*\t(\d*)\t\d*\t\d*\t\d*/ )
		{
			$ssflag++ unless $1 > 0;
			if ( $linecount == $exstart )
			{
				$revend = $1;
			}
			if ( $linecount == $sstart )
			{
				$revendbackup = $1;
			}
			if ( $linecount == $send - 2)
			{
				$revstart = $1;
			}
			if ( $linecount == $send )
			{
				$revstartbackup = $1;
			}
		}
	}
	close FOLD;
	if ( $revend == 0 )
		{
			$revend = $revendbackup - 2;
		}
	if ( $revstart == 0 )
		{
			$revstart = $revstartbackup - 2;
		}
	$revlength = $revend - $revstart + 1;
	if ( $ssflag > 4 )
	{
		print LOGFILE "Too many unpaired bases in the miRNA region of $sid.
Files deleted.\n";
		print DISCARD "$sid\t$qid\t$sstart\t$send\t$ssflag unpaired bases
in miRNA\n";
		system ("rm $uniqueid.*");
		$badcount++;
	}
	elsif ( $revend == -2 or $revstart == -2)
```

```
	{
		print LOGFILE "One end of the miRNA region was not base-paired, so 
could not locate the end of the miRNA complementary sequence.  This hit will be 
placed in the 'suspect' table.\n";
		if ( $revend == -2 )
		{
			$revend = $revstart + $qlength - 1;
		}
		elsif ( $revstart == -2 )
		{
			$revstart = $revend - $qlength + 1;
		}
		print SUSPECT 
"$uniqueid\t$sid\t$qid\t$qlength\t$sstart\t$send\t$revstart\t$revend\n";
		open ( FASTA, $uniqueid.".fsa");
		while ($line = <FASTA>)
		{
			chomp $line;
			if ( $line =~ /^>/ )
			{
				print SUSPECTOUT "$uniqueid\t";
			}
			else
			{
				print SUSPECTOUT "$line";
			}
		}
		print SUSPECTOUT "\n";
		close FASTA;
		system ("rm $uniqueid.fsa.*");
		system ("rm $uniqueid.fsa_1.ss");
		system ("rm $uniqueid.fsa_1.pdf");
		system ("mv $uniqueid.fsa $infile.initialfolds");
		$suspectcount++;
	}
	elsif ( $revlength - $qlength > 3 )
	{
		print LOGFILE "The miRNA complementary sequence of $sid contains 
breaks or a large loop. Files deleted.\n";
		print DISCARD "$sid\t$qid\t$sstart\t$send\tmiRNA complementary 
sequence is broken \n";
		system ("rm $uniqueid.*");
		$badcount++;
	}
	elsif ( $ssflag == 0 )
	{
		print LOGFILE "The putative miRNA sequence of $sid is perfectly 
base-paired, so it is more likely to be an inverted repeat or siRNA.  This hit 
will be placed in the 'suspect' table.\n";
		print SUSPECT 
"$uniqueid\t$sid\t$qid\t$qlength\t$sstart\t$send\t$revstart\t$revend\n";
		open ( FASTA, $uniqueid.".fsa");
		while ($line = <FASTA>)
		{
			chomp $line;
			if ( $line =~ /^>/ )
			{
				print SUSPECTOUT "$uniqueid\t";
			}
			else
			{
```

```
				print SUSPECTOUT "$line";
			}
		}
		print SUSPECTOUT "\n";
		close FASTA;
		system ("rm $uniqueid.fsa.*");
		system ("rm $uniqueid.fsa_1.ss");
		system ("rm $uniqueid.fsa_1.pdf");
		system ("mv $uniqueid.fsa $infile.initialfolds");
		$suspectcount++;
	}
	else
	{
		print LOGFILE "The secondary structure of $uniqueid passes initial
analysis, writing to output table and fasta file.\n";
		print OUTTABLE
"$uniqueid\t$sid\t$qid\t$qlength\t$sstart\t$send\t$revstart\t$revend\n";
		open ( FASTA, $uniqueid.".fsa");
		while ($line = <FASTA>)
		{
			chomp $line;
			if ( $line =~ /^>/ )
			{
				print OUT "$uniqueid\t";
			}
			else
			{
				print OUT "$line";
			}
		}
		print OUT "\n";
		close FASTA;
		system ("rm $uniqueid.fsa.*");
		system ("rm $uniqueid.fsa_1.ss");
		system ("rm $uniqueid.fsa_1.pdf");
		system ("mv $uniqueid.fsa $infile.initialfolds");
		$goodcount++;
	}
}
close IN;
close OUT;
close OUTTABLE;
close SUSPECT;
close SUSPECTOUT;

# Shunt RNA secondary structures into a folder

system ("mv *.fsa_1.* $infile.initialfolds");

print LOGFILE "From the input table, $goodcount sequence(s) gave folds that could
contain a miRNA.\n$badcount sequence(s) were rejected after
folding.\n$suspectcount sequence(s) are more likely to be repeats or siRNAs.\n\n";
close LOGFILE;

# Conduct further analysis on hairpin regions of all good hits

open ( GOODTABLE, $infile.".tbl" );
open ( RESULTS, ">".$infile.".hairpins.tbl" ) or die "Can't open an output
file!\n";
open ( HAIRPINS, ">".$infile.".hairpins.fsa" ) or die "Can't open an output
file!\n";
```

```
open ( LOGFILE2, ">".$infile.".hairpins.log" ) or die "Can't open an output
file!\n";
print RESULTS "Unique\tNew miRNA\t\t\tConserved
miRNA\t\t\tSequence\tMature\tMature\tHairpin\tPre-miRNA stats\n";
print RESULTS "Hit
ID\tID\tSequence\tLength\tID\tSequence\tMismatch\tID\tStart\tEnd\tlocation\tlength
\tMFE\tGC%\tMFEI\tstart\tsequence\n";
my $goodhairpins = 0;

while ( $line = <GOODTABLE> )
{
	chomp $line;
	next if $line =~ /^#/;
	my ( $uniqueid, $sseqid, $mirnaid, $length, $sstart, $send, $revstart,
$revend ) = split /\t/, $line;
	my ( $armflag, $seq ) = "";
	my ( $hairpinstart, $hairpinend, $hairpinlength ) = 0;
	print LOGFILE2 "Analysing hit $uniqueid for $mirnaid\n";

# Determine which part of the sequence corresponds to putative hairpin and
retrieve it from the sequence table
	if ( $sstart > $revstart )
	{
		$armflag = "3'";
		$hairpinstart = $revstart - 20;
		$hairpinend = $send + 20;
	}
	elsif ( $sstart < $revstart )
	{
		$armflag = "5'";
		$hairpinstart = $sstart - 20;
		$hairpinend = $revend + 20;
	}
	else
	{
		print LOGFILE2 "The miRNA co-ordinates for $uniqueid don't make
sense!  Skipping it.\n";
		next;
	}

	open ( GOODSEQS, $infile.".seqtable" );
	while ( $line = <GOODSEQS> )
	{
		my ( $fseqid, $fseq) = split /\t/, $line;
		if  ( $fseqid =~ m/$uniqueid/ )
		{
			$seq = $fseq;
			last;
		}
	}
	close GOODSEQS;
	if ( $hairpinend - $hairpinstart < (2*$length) + 40 )
	{
		print LOGFILE2 "The miRNA region of $sseqid goes round the head of
the hairpin; discarded.\n";
		print DISCARD "$sseqid\t$mirnaid\t$sstart\t$send\tmiRNA goes round
head of hairpin\n";
		next;
	}
	if ( $hairpinstart < 1 )
	{
```

```
                $hairpinstart = 1;
        }
        if ( $hairpinend > length $seq )
        {
                $hairpinend = length $seq;
        }
        $hairpinlength = $hairpinend - $hairpinstart + 1;

        my $hairpinseq = substr $seq, ($hairpinstart-1), $hairpinlength;
        my $matseq = substr $seq, ($sstart-1), $length;
        my $matstart = $sstart - $hairpinstart;
        my $matend = $send - $hairpinstart;
        print LOGFILE2 "Hairpin stats (start, end, length):\n";
        print LOGFILE2 "$hairpinstart\t$hairpinend\t$hairpinlength\n";
        print LOGFILE2 "Running UNAFold.pl on the hairpin sequence... ";
        open ( PFASTA, ">".$uniqueid.".hairpin.fsa" ) or die "Can't open an output
file!\n";
        print PFASTA ">$sseqid\t$mirnaid\n";
        print PFASTA "$hairpinseq\n";
        close PFASTA;

# Run UNAFold on the hairpin, get useful information from the .ct file

        system ("perl /usr/bin/UNAFold.pl -X 1 --ann ss-count
$uniqueid.hairpin.fsa");
        open ( HAIRPINFOLD, $uniqueid.".hairpin.fsa.ct" ) or die "where is the ct
file?";
        my $GCcount = 0;
        while ( $line = <HAIRPINFOLD> )
        {
                chomp $line;
                if ( $line =~ m/dG =\s(\S+)/ )
                {
                        $mfe = $1;
                }
                elsif ( $line =~ /\d*\t(\w)\t\d*\t\d*\t\d*\t\d*\t\d*\t\d*/ )
                {
                        $GCcount++ if $1 =~ /(G|C)/;
                }
        }
        close HAIRPINFOLD;

# Process data and print results files

        my $mirnafam = substr ($mirnaid, 3);
        my $conseq = $QuerymiRNAs{"$mirnaid"};
        my $GCcomp = 100*$GCcount/$hairpinlength;
        my $amfe = (0-100*$mfe/$hairpinlength);
        my $mfei = $amfe/$GCcomp;
        my $mism = 0;
        for ($loopcount = 0; $loopcount < $length; $loopcount++)
        {
                $mism++ if substr ($conseq, $loopcount, 1) ne substr ($matseq,
$loopcount, 1);
        }
        if ( $GCcomp < 24 or $GCcomp > 71 )
        {
                print LOGFILE2 "The hairpin for $uniqueid has too low or high GC
content.  Deleting files.\n";
                print DISCARD "$sseqid\t$mirnaid\t$sstart\t$send\tGC content
outside acceptable range\n";
```

```
                system ("rm $uniqueid.*");
        }
        elsif ( $mfei < 0.67 )
        {
                print LOGFILE2 "The hairpin for $uniqueid has MFEI < 0.67.
Deleting files.\n";
                print DISCARD "$sseqid\t$mirnaid\t$sstart\t$send\tMFEI outside
acceptable range\n";
                system ("rm $uniqueid.*");
        }
        else
        {
                print LOGFILE2 "The hairpin passes analysis, printing to results
table\n";
                print RESULTS
"$uniqueid\ttae$mirnafam\t$matseq\t$length\t$mirnaid\t$conseq\t$mism\t$sseqid\t$ma
tstart\t$matend\t$armflag\t$hairpinlength\t$mfe\t$GCcomp\t$mfei\t$hairpinstart\t$h
airpinseq\n";
                print HAIRPINS ">$sseqid\t$mirnaid\n$hairpinseq\n";
                system ("rm $uniqueid.hairpin.fsa.*");
                system ("rm $uniqueid.hairpin.fsa_1.ss");
                system ("rm $uniqueid.hairpin.fsa_1.pdf");
                system ("mv $uniqueid.hairpin.fsa $infile.hairpins");
                $goodhairpins++
        }
}
close RESULTS;
close HAIRPINS;
close GOODTABLE;

# Carry out the same hairpin analysis on suspect hits
print LOGFILE2 "Moving on to examine suspect hits from initial folding
analysis...\n";
open ( SUSPECTTABLE, $infile.".suspect.tbl" );
open ( SUSPECTRESULTS, ">".$infile.".suspecthairpins.tbl" ) or die "Can't open an
output file!\n";
open ( SUSPECTHAIRPINS, ">".$infile.".suspecthairpins.fsa" ) or die "Can't open an
output file!\n";
print SUSPECTRESULTS "Unique\tNew miRNA\t\t\tConserved
miRNA\t\t\tSequence\tMature\tMature\tHairpin\tPre-miRNA stats\n";
print SUSPECTRESULTS "Hit
ID\tID\tSequence\tLength\tID\tSequence\tMismatch\tID\tStart\tEnd\tlocation\tlength
\tMFE\tGC%\tMFEI\tstart\tsequence\n";
my $suspecthairpins = 0;

while ( $line = <SUSPECTTABLE> )
{
        chomp $line;
        next if $line =~ /^#/;
        my ( $uniqueid, $sseqid, $mirnaid, $length, $sstart, $send, $revstart,
$revend ) = split /\t/, $line;
        my ( $armflag, $seq ) = "";
        my ( $hairpinstart, $hairpinend, $hairpinlength ) = 0;
        print LOGFILE2 "Analysing hit $uniqueid\n";

# Determine which part of the sequence corresponds to putative hairpin and
retrieve it from the sequence table
        if ( $sstart > $revstart )
        {
                $armflag = "3'";
                $hairpinstart = $revstart - 20;
```

```
		$hairpinend = $send + 20;
	}
	elsif ( $sstart < $revstart )
	{
		$armflag = "5'";
		$hairpinstart = $sstart - 20;
		$hairpinend = $revend + 20;
	}
	else
	{
		print LOGFILE2 "The miRNA co-ordinates for $uniqueid don't make 
sense!  Skipping it.\n";
		next;
	}

	open ( SUSPECTSEQS, $infile.".suspect.seqtable" );
	while ( $line = <SUSPECTSEQS> )
	{
		my ( $fseqid, $fseq) = split /\t/, $line;
		if  ( $fseqid =~ m/$uniqueid/ )
		{
			$seq = $fseq;
			last;
		}
	}
	close SUSPECTSEQS;
	if ( $hairpinend - $hairpinstart < (2*$length) + 40 )
	{
		print LOGFILE2 "The miRNA region of $sseqid goes round the head of 
the hairpin; discarded.\n";
		print DISCARD "$sseqid\t$mirnaid\t$sstart\t$send\tmiRNA goes round 
head of hairpin\n";
		next;
	}
	if ( $hairpinstart < 1 )
	{
		$hairpinstart = 1;
	}
	if ( $hairpinend > length $seq )
	{
		$hairpinend = length $seq;
	}
	$hairpinlength = $hairpinend - $hairpinstart + 1;

	my $hairpinseq = substr $seq, ($hairpinstart-1), $hairpinlength;
	my $matseq = substr $seq, ($sstart-1), $length;
	my $matstart = $sstart - $hairpinstart;
	my $matend = $send - $hairpinstart;
	print LOGFILE2 "Hairpin stats (start, end, length):\n";
	print LOGFILE2 "$hairpinstart\t$hairpinend\t$hairpinlength\n";
	print LOGFILE2 "Running UNAFold.pl on the hairpin sequence... ";
	open ( PFASTA, ">".$uniqueid.".hairpin.fsa" ) or die "Can't open an output 
file!\n";
	print PFASTA ">$sseqid\t$mirnaid\n";
	print PFASTA "$hairpinseq\n";
	close PFASTA;

# Run UNAFold on the hairpin, get useful information from the .ct file

	system ("perl /usr/bin/UNAFold.pl -X 1 --ann ss-count 
$uniqueid.hairpin.fsa");
```

```
        open ( HAIRPINFOLD, $uniqueid.".hairpin.fsa.ct" ) or die "where is the ct
file?";
        my $GCcount = 0;
        while ( $line = <HAIRPINFOLD> )
        {
                chomp $line;
                if ( $line =~ m/dG =\s(\S+)/ )
                {
                        $mfe = $1;
                }
                elsif ( $line =~ /\d*\t(\w)\t\d*\t\d*\t\d*\t\d*\t\d*\t\d*/ )
                {
                        $GCcount++ if $1 =~ /(G|C)/;
                }
        }
        close HAIRPINFOLD;

# Process data and print results files

        my $mirnafam = substr ($mirnaid, 3);
        my $conseq = $QuerymiRNAs{"$mirnaid"};
        my $GCcomp = 100*$GCcount/$hairpinlength;
        my $amfe = (0-100*$mfe/$hairpinlength);
        my $mfei = $amfe/$GCcomp;
        my $mism = 0;
        for ($loopcount = 0; $loopcount < $length; $loopcount++)
        {
                $mism++ if substr ($conseq, $loopcount, 1) ne substr ($matseq,
$loopcount, 1);
        }
        if ( $GCcomp < 24 or $GCcomp > 71 )
        {
                print LOGFILE2 "The hairpin for $uniqueid has too low or high GC
content.  Deleting files.\n";
                print DISCARD "$sseqid\t$mirnaid\t$sstart\t$send\tGC content
outside acceptable range\n";
                system ("rm $uniqueid.*");
        }
        elsif ( $mfei < 0.67 )
        {
                print LOGFILE2 "The hairpin for $uniqueid has MFEI < 0.67.
Deleting files.\n";
                print DISCARD "$sseqid\t$mirnaid\t$sstart\t$send\tMFEI outside
acceptable range\n";
                system ("rm $uniqueid.*");
        }
        else
        {
                print LOGFILE2 "The hairpin passes analysis, printing to suspect
results table\n";
                print SUSPECTRESULTS
"$uniqueid\ttae$mirnafam\t$matseq\t$length\t$mirnaid\t$conseq\t$mism\t$sseqid\t$ma
tstart\t$matend\t$armflag\t$hairpinlength\t$mfe\t$GCcomp\t$mfei\t$hairpinstart\t$h
airpinseq\n";
                print SUSPECTHAIRPINS ">$sseqid\t$mirnaid\n$hairpinseq\n";
                system ("rm $uniqueid.hairpin.fsa.*");
                system ("rm $uniqueid.hairpin.fsa_1.ss");
                system ("rm $uniqueid.hairpin.fsa_1.pdf");
                system ("mv $uniqueid.hairpin.fsa $infile.hairpins");
                $suspecthairpins++
        }
```

```
}


print LOGFILE2 "\nFrom $goodcount viable folds, $goodhairpins passed the hairpin
statistics criteria.\n";
print LOGFILE2 "From $suspectcount suspect but possible folds, $suspecthairpins
passed the hairpin statistics criteria.\n";
system ("mv *.fsa_1.* $infile.hairpins");
close SUSPECTTABLE;
close SUSPECTHAIRPINS;
close SUSPECTRESULTS;
close DISCARD;
close LOGFILE2;
```

These scripts are free software: you can redistribute it and/or modify them under the terms of the GNU General Public License as published by the Free Software Foundation, version 3 of the License.

These scripts are distributed in the hope that they will be useful, but WITHOUT ANY WARRANTY; without even the implied warranty of MERCHANTABILITY or FITNESS FOR A PARTICULAR PURPOSE. See the GNU General Public License for more details: http://www.gnu.org/licenses/
